# Ozone Overload: Current Standards May Not Protect Health

**Published:** 2006-04

**Authors:** Richard Dahl

Ozone is a common urban pollutant that has been linked to health effects such as reduced lung function, increases in respiratory symptoms, and development of asthma. Now a team of researchers reports that ozone may pose a danger to human health even at levels far below the limits set by current U.S. and international regulations **[*EHP* 114:532–536; Bell et al.]**. The team conducted a study of 98 U.S. urban communities between 1987 and 2000 to investigate whether there is a threshold below which ozone does not affect mortality, and report that they were unable to identify such a threshold.

More than 100 million Americans live in areas that exceed the EPA’s National Ambient Air Quality Standard (NAAQS) for ozone of 80 parts per billion (ppb) ozone averaged over a peak 8-hour time period. The EPA is currently reviewing scientific evidence to determine whether that NAAQS should be revised in order to meet the 1997 Clean Air Act’s goal of protecting human health with an adequate margin of safety.

The researchers embarked on this project to better identify that margin. Data were gathered from the National Morbidity, Mortality, and Air Pollution Study, a project launched in 1996 to address questions about the degree to which particulate matter is responsible for changes in daily mortality rates. They also used ozone data from the EPA and weather data from the National Climatic Data Center. Then they applied a Bayesian hierarchical model to mortality data from the National Center for Health Statistics to evaluate the relationship between ambient ozone levels and mortality rates within each community over a 14-year period.

The key finding of their research is strong and consistent evidence that daily increases in ambient ozone exposure were associated with daily increases in premature mortality. This was true even at very low pollution levels, including an idealized scenario in which every community always met current ozone regulations. In that scenario, each daily 10-ppb increase of 8-hour ozone was associated with a 0.30% increase in mortality.

“All results indicate that any threshold would exist at very low concentrations, far below current U.S. and international regulations and nearing background levels,” the authors write. They conclude that any reduction in ambient ozone levels, such as through transportation planning in urban areas, could be expected to yield important benefits to public health, even in areas that already meet current regulatory standards.

## Figures and Tables

**Figure:**
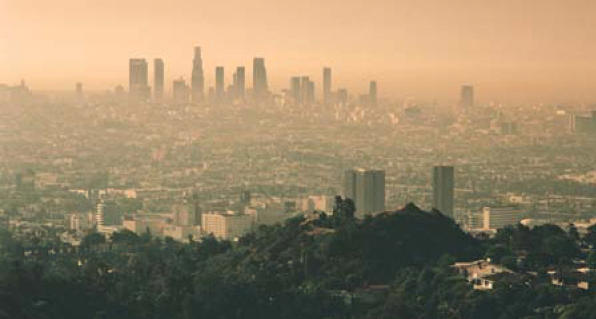
Smog alert. A new study was unable to identify a threshold below which ozone no longer affected premature mortality.

